# Difference in R01 Grant Funding Among Osteopathic and Allopathic Emergency Physicians over the Last Decade

**DOI:** 10.5811/westjem.2017.1.32964

**Published:** 2017-04-17

**Authors:** Martina Antony, Jennifer Savino, John Ashurst

**Affiliations:** Duke Lifepoint Conemaugh Memorial Medical Center, Department of Emergency Medicine, Johnstown, Pennsylvania

## Abstract

**Introduction:**

Receiving an R01 grant from the National Institutes of Health (NIH) is regarded as a major accomplishment for the physician researcher and can be used as a means of scholarly activity for core faculty in emergency medicine (EM). However, the Accreditation Council for Graduate Medical Education requires that a grant must be obtained for it to count towards a core faculty member’s scholarly activity, while the American Osteopathic Association states that an application for a grant would qualify for scholarly activity whether it is received or not. The aim of the study was to determine if a medical degree disparity exists between those who successfully receive an EM R01 grant and those who do not, and to determine the publication characteristics of those recipients.

**Methods:**

We queried the NIH RePORTER search engine for those physicians who received an R01 grant in EM. Degree designation was then determined for each grant recipient based on a web-based search involving the recipient’s name and the location where the grant was awarded. The grant recipient was then queried through PubMed central for the total number of publications published in the decade prior to receiving the grant.

**Results:**

We noted a total of 264 R01 grant recipients during the study period; of those who received the award, 78.03% were allopathic physicians. No osteopathic physician had received an R01 grant in EM over the past 10 years. Of those allopathic physicians who received the grant, 44.17% held a dual degree. Allopathic physicians had an average of 48.05 publications over the 10 years prior to grant receipt and those with a dual degree had 51.62 publications.

**Conclusion:**

Allopathic physicians comprise the majority of those who have received an R01 grant in EM over the last decade. These physicians typically have numerous prior publications and an advanced degree.

## INTRODUCTION

For many physician researchers, receiving the National Institutes of Health (NIH) R01 grant is a major accomplishment and serves as an early career milestone from which further granting opportunities arise. However, receiving one of these prestigious grants is a rarity in academic emergency medicine (EM). A 2011 study found that 18 investigators in 2010 had one of these grants despite the relatively large population of practicing emergency physicians.^1^

In that study, however, there was no distinction made with regard to what type of degree the physician researcher held. Recently, a disparity has been noted among osteopathic and allopathic physicians who serve on editorial boards and publish manuscripts in EM.^2^,^3^ With the recent merger of the Accreditation Council for Graduate Medical Education (ACGME) and the American Osteopathic Association (AOA), a heightened awareness of these topics may become evident. The ACGME states that obtaining a grant can be applied to a core faculty member’s scholarly activity, while the AOA notes that applying for a grant can meet this requirement.^4^,^5^ In the current study, we looked to assess if there was a difference in R01 grants being awarded among the different medical professions in EM.

## METHODS

### Study Design

After obtaining institution review board approval, we queried the NIH RePORTER search engine (http://projectreporter.nih.gov) for “emergency medicine” as a key word for 2006 through 2016 for R01 grants. Each recipient was then categorized as either having a degree in osteopathic or allopathic medicine. We did this by completing a web-based search for the author and the author’s home institution. Secondarily, we recorded any advanced degrees for each grant recipient.

Once the grant recipient was determined to be accurate, we quantified the author’s publication activity by determining the number of peer-reviewed manuscripts published by the recipient in the10 years prior to being awarded the grant. This was accomplished by completing a PubMed (http://www.pubmed.com) search using the author’s name. The author was then classified as either the first, second or last author for each manuscript.

We analyzed comparison of the proportions of allopathic and osteopathic physician grant recipients across the years by using simple descriptive statistics. The percentage of those holding both a medical degree and an advanced degree was also analyzed using descriptive statistics.

## RESULTS

We identified a total of 264 R01 grant recipients during the study period. Allopathic physicians accounted for 78.03% (206/264) of all grant recipients, and no osteopathic physicians were awarded an EM R01 grant during the study period. Those who were categorized in the “other degree” category comprised the remainder of the grant recipients. Of the allopathic physicians awarded an EM R01 grant, 44.17% (91/206) held a dual advanced degree.

In the 10 years preceding the receipt of their award, all recipients had prior research publications that were identified in PubMed. Allopathic physicians accounted for 79.32% (2,881/3,758) of the primary authors, 73.31% (1,173/1,600) of secondary authors and 73.80% (5,744/7,783) of all senior authors. On average, each allopathic physician who successfully obtained an R01 grant had 48.05 publications over the 10-year period prior to receiving the grant. Upon subgroup analysis of allopathic physicians, we found that those with a dual degree comprised 52.90% (1,577/2,981) of primary authors, 41.69% (488/1173) of secondary authors and 45.82% (2,632/5,744) of all senior authors. Allopathic physicians who held a dual degree had on average 51.62 publications in the 10 years prior to receiving their R01 grants.

## DISCUSSION

This is the first article to describe a medical degree disparity between those physicians who receive EM R01 grants and those who do not. Over the last 10 years, the majority of recipients of an EM R01 grant have been allopathic physicians, and no osteopathic physician has received an EM R01 grant during that same period. It is unclear why this disparity exists, but it appears that prior research publications and advanced research training play a crucial role in determining who receives EM R01 grants.

Recent literature has shown that there is a disparity in medical degree designation among those who have published manuscripts in high-ranking EM journals over the last two decades. According to Lammers et al., very few osteopathic EM physicians are either the first or senior author on original research manuscripts in these journals.^3^ Previous data collected on those who have received EM R01 grants has shown that recipients were publishing approximately five articles a year and had published 38 peer-reviewed manuscripts.^1^ The current data also has shown that almost 75% of all allopathic recipients have served in the role of first, second and senior author on numerous manuscripts. Based upon the prior results, coupled with the data found by this study, it appears that prior publications are a key component in determining who will be awarded a NIH R01 grant in EM. Without a track record of prior publications, osteopathic emergency physicians are at a disadvantage with regard to being awarded an R01 grant.

The current study also shows that almost half of all allopathic physicians who have received an EM R01 grant hold an advanced degree. A dual-degree program offers the physician researcher the opportunity to hone his/her research skills and work directly with a mentor who may have already successfully navigated the granting process. A total of 26 osteopathic medical students were enrolled in a dual-degree program in 2004 as reported by the American Association of Colleges of Osteopathic Medicine.^6^ It is unclear which specialty these students chose to practice medicine in; however, it would make up the minority of practicing osteopathic physicians regardless. Without this prior training in the rigors of academic medicine, it is difficult for a community-based physician to be awarded an R01 grant.

Previous reported data has also shown that the median age to receive an R01 grant in EM was 43 years.^1^ Based upon the previous data, coupled with the lack of osteopathic physician researchers with a dual degree, it can be theorized that the osteopathic physician researchers who hold a dual degree have not yet reached a point in their careers where they would feel qualified to apply for an R01 grant.,

It has been previously noted that receiving an R01 grant from the NIH is a gateway to increased involvement in peer review at the national level and is regarded as a seminal event for the physician researcher.^1^ Since the majority of those receiving this grant are allopathic physicians, the views of osteopathic emergency physicians may not be expressed at these national levels. This has been evidenced by the lack of osteopathic physicians who have served on the editorial boards of major academic journals including *Annals of Emergency Medicine*.^3^

A core faculty member’s scholarly activity is a key component in determining if a residency program receives a citation from either the AOA or ACGME upon its site review. The current results show that no osteopathic EP has obtained a NIH R01 grant over the last decade, and based upon the new single accreditation standards would not have received any credit for scholarly activity. Previously, however, applying for a grant would have awarded an osteopathic core faculty member scholarly activity. As progression occurs through the merger, osteopathic EM residencies must be aware of these changes and attempt to rectify the situation through increased faculty development in order to develop faculty members who are better candidates for national grants.

## LIMITATIONS

There are several key limitations to this study. First, the authors only reviewed one specialty’s receipt of R01 grants over the last decade. Other specialties may have had more of an osteopathic presence among those who received R01 grants, which may alter the conclusions we have drawn. Also, the authors used a web-based search to determine the grant recipients’ degree designations. Although the author’s home institution was examined, the data on the website may not have been accurate and would therefore alter the results. We also note that PubMed is unable to distinguish the difference between those physicians who have the same name. This may have led to an increased number of publications being reported. Similarly, PubMed does not include all journals that are currently being published and the authors may have published manuscripts in journals that are not in the PubMed repository. Because we were unable to comment on the years that were not reviewed, it is possible that inclusion of a greater time span might have shown osteopathic physician-researcher involvement.

## CONCLUSION

Authors who have received an R01 grant from the NIH in emergency medicine are primarily allopathic physicians who hold a dual degree and have a track record of prior publications. The osteopathic community must continue to further educate physician researchers in order to close the medical degree disparity gap among those who receive grant funding from the NIH.
